# Association of Case Volume With Ablation Outcomes in Children: Analysis of the NCDR IMPACT Registry

**DOI:** 10.1016/j.jacadv.2026.102846

**Published:** 2026-06-17

**Authors:** Dustin A. Nash, Kevin F. Kennedy, Maully Shah, V Ramesh Iyer, Tammy Sweeten, Chandra Srinivasan, Michael L. O’Byrne, Christopher M. Janson

**Affiliations:** a Division of Cardiology, Department of Pediatrics, Children’s Hospital Colorado and University of Colorado School of Medicine, Aurora, Colorado, USA; b Mid America Heart Institute and St. Luke's Health System, Kansas City, Missouri, USA; c Division of Cardiology, The Children's Hospital of Philadelphia, Philadelphia, Pennsylvania, USA; d Department of Pediatrics, Perelman School of Medicine at the University of Pennsylvania, Philadelphia, Pennsylvania, USA

**Keywords:** catheter ablation, center volume, operator volume, pediatrics

## Abstract

**Background:**

Increasing case volume has been positively associated with procedural outcomes in many fields, but little is known about this relationship for pediatric catheter ablation.

**Objectives:**

The objective of the study was to report associations between center and operator case volumes and acute success in pediatric ablations.

**Methods:**

A multicenter retrospective cohort study of patients undergoing ablation at Improving Pediatric And Adult Congenital Treatment registry sites from April 2016-March 2020 was performed evaluating the association between center or operator annual case volume (low, medium, high, or very high) and the likelihood of acute ablation success. Unadjusted comparisons and multivariable logistic regression was completed with subgroup analyses to evaluate associations in higher risk subgroups.

**Results:**

Of 18,666 ablations at 74 hospitals, acute success was reported in 17,204 (92%). High/very high-volume centers showed marginal but statistically insignificant increases in the rates of acute success when compared to medium/low centers (92% vs 91%; *P* = 0.24). In multivariable models high volume centers saw increased odds of success in patients with congenital heart disease (OR: 2.3; CI: 1.21-4.4), and patients with premature ventricular contractions/ventricular tachycardia (OR: 2.2; CI: 1.23-4.1). Among 156 operators, the highest rates of acute success were seen in very high-volume operators (94%) compared to low (92.4%), medium (91.9%), and high (92.2%) operators (*P* = 0.015). Multivariable modeling showed no association between operator volume and acute success.

**Conclusions:**

Pediatric catheter ablation acute success was high across a range of center and operator volumes. Complex substrates have increased odds of success at high volume centers, and high and very high-volume centers have consistent rates of success despite increasing case complexity.

Catheter ablation therapy is a cornerstone of treatment for children and adolescents with cardiac arrhythmias, demonstrating both safety and efficacy.^1^^,^^2^ Recent data indicate that up to 10% of pediatric ablation cases do not result in acute success,^3^ identifying a target for outcome improvement. Higher procedural volume has been linked to lower mortality in congenital heart surgery^4^, ^5^, ^6^, ^7^, ^8^, ^9^, ^10^, ^11^, ^12^, ^13^ and fewer major adverse events in congenital cardiac catheterization.^14^, ^15^, ^16^, ^21^ Similarly, low center volume was associated with increased likelihood of adverse outcomes in 1 study of atrial fibrillation ablation procedures in adults.^17^ To our knowledge, the association(s) between center and operator volume and the likelihood of procedural success and adverse outcomes in pediatric ablations is not well understood.

Studying this question is challenging. First, adverse events are rare during pediatric ablation (<1% of cases).^1^^,^^2^ Second, the likelihood of both technical success and adverse events are affected by patient characteristics (age, size, and congenital heart disease [CHD]) and ablation substrate (Wolff-Parkinson-White [WPW], vs ventricular tachycardia [VT] vs ectopic atrial tachycardia). To overcome these limitations, many cases from a variety of centers is necessary, data which until recently were not available. We sought to overcome these limitations by performing a retrospective cohort study using data from the IMPACT (Improving Pediatric and Adult Congenital Treatment) registry, a database of U.S. pediatric/congenital electrophysiology procedures, which provides a contemporary and representative sample at a national scale. We hypothesized that procedural success will be higher and adverse event rates will be lower at the highest volume programs relative to lower volume programs (Central Illustration).

**Central Illustration:**
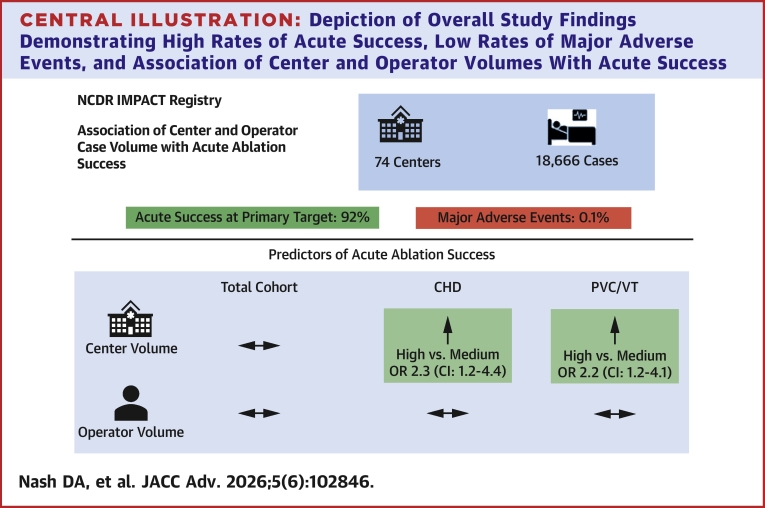
**Depiction of Overall Study Findings Demonstrating High Rates of Acute Success, Low Rates of Major Adverse Events, and Association of Center and Operator Volumes With Acute Success** CHD = congenital heart disease; IMPACT = Improving Pediatric and Adult Congenital Treatment; NCDR = National Cardiovascular Data Registry; PVC = premature ventricular contraction; VT = ventricular tachycardia.

## Methods

### Data source

IMPACT is a registry funded by the American College of Cardiology Foundation and managed by the National Cardiovascular Data Registry.^18^ It includes demographic, medical/surgical history, and procedural information from 98 centers where pediatric/congenital cardiac catheterization and electrophysiology procedures are performed. Data are recorded in a standardized format using previously established data definitions and are subject to quality assurance standards.^1^ The current study used data from IMPACT v2.

This study used deidentified patient, center, and operator data and is exempt from institutional review board review in accordance with the Common Rule (45 CFR 46.102[f]). Due to data use agreements of the contributing centers with IMPACT, no subject-level data can be shared. Statistical methods will be shared on request.

### Study design and cohort

We performed a multicenter retrospective cohort study of catheter ablation cases in children and adolescents (age ≤21 years) at centers entering data into IMPACT from April 1, 2016, through April 1, 2020. Considering the complicated relationship between center and operator volume, we conducted parallel analyses to independently evaluate center volume and operator volume.

We designed our analysis to sample from centers and operators primarily dedicated to pediatric catheter ablation with enough longitudinal data to draw meaningful conclusions. To this end, we excluded cases from centers or operators contributing fewer than 5 quarters of data to IMPACT, those with a high proportion of diagnostic electrophysiology studies without ablation (>30%), or a high proportion of adult cases (>40% of patients >18 years of age). These cutoffs were selected based on visual inspection of the data. Of 98 centers contributing data on 20,839 ablations during the study period, 24 centers were excluded from the analysis of center volume (Supplemental Figure 1). Of the 428 operators, we excluded 303 operators from our analysis of operator volume (Supplemental Figure 2). This notably high exclusion rate was explained by 226 (52%) of the operators having <5 quarters of data with over 90% having submitted <10 cases to the registry and the remaining exclusions for providers high rates of diagnostic studies and adult patients. We excluded cases that were outliers in terms of complexity and/or rarity, including cases with >3 ablation targets, atriofascicular pathways (unidirectional antegrade decremental pathways), AV nodal ablations, fast pathway ablations, or sinus node modifications.

### Study measures

Deidentified demographic and procedural data were extracted from the IMPACT database. Two parallel analyses were performed, 1 with a primary exposure of center case volume and 1 with a primary exposure of operator volume. Annual case volume, representing an average annual volume during the study period and subsequently referred to throughout this manuscript simply as volume, was calculated for each center or operator as the sum of all cases, divided by the number of quarters of data available for that center or operator, and then multiplied by four. As in previous studies, average case volume over the study period was used because it is more representative of the experience of the center or operator than those implied by year-to-year changes.

**Figure 1:**
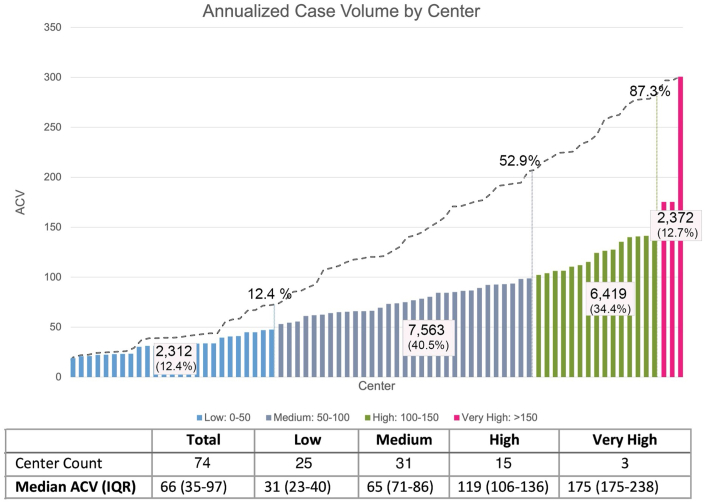
**Annualized Case Volume by Center** Histogram sorted by annualized case volume color coded by case volume class. The dashed line represents the total volume of cases and inset over each class is the total volume for each class and the percentage of total cases performed by that class. The subset table shows the number of centers by volume class as well as their median annualized case volume (ACV).

After applying exclusion criteria, 74 centers with 18,666 unique ablations cases remained for the analysis of center volume. Centers were sorted by case volume from lowest to highest and then visually inspected to define quartiles. There were incremental increases in case volume up to a value of 150 cases per year, followed by a noticeable jump for the last 3 centers (Figure 1). Based on the observed distributions, case volume was defined as follows: low <50 cases per year; medium as 50 to 100 cases per year; high as 101 to 150 cases per year; and very high as >150 cases per year. There were 25 (33%) low, 33 (41%) medium, 15 (20%) high, and 3 (4%) very high volume centers.

**Figure 2:**
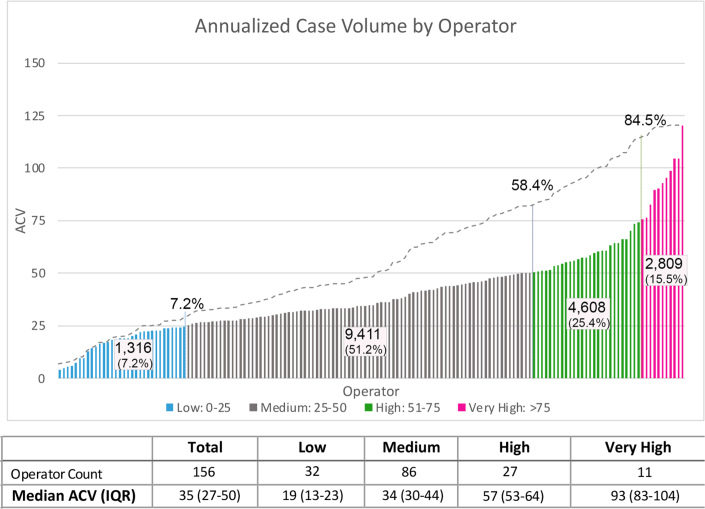
**Annualized Case Volume by Operator** Histogram sorted by annualized case volume color coded by case volume class. The dashed line represents the total volume of cases and inset over each class is the total volume for each class and the percentage of total cases performed by that class. The subset table shows the number of centers by volume class as well as their median annualized case volume (ACV).

After applying exclusion criteria, 156 operators with 18,144 unique ablations cases remained for the analysis of operator volume. As previously, operators were sorted by case volume from lowest to highest and the data then visually inspected. The operator case volume curve had a sigmoidal shape with inflection points at case volume values of 25, 50, and 75 (Figure 2). Annualized volumes were classified as: low <25 cases per year; medium as 25 to 50 cases per year; high as 51 to 75 cases per year; and very high as >75 cases per year. There were 32 (21%) low, 86 (55%) medium 27 (17%) high, and 11 (7%) very high volume operators.

The primary outcome was acute ablation success at the primary target. This primary outcome was selected rather than adverse events as previously applied in congenital cardiac catheterization literature, given the very low rate of ablation complications reported in IMPACT studies.^19^ Substrate-specific definitions of acute ablation success are detailed in Supplemental Table 1. Secondary outcomes included acute ablation success at secondary and tertiary targets, acute ablation success at all targets, procedural duration, any adverse event, and major adverse events (MAE). MAE were defined to include death, cardiac arrest, heart block requiring permanent pacemaker, embolic stroke, tamponade, or need for urgent cardiac/vascular surgery.

Potential covariates were included based on previously published correlates of ablation success.^20^ These included the following: 1) patient age, divided into the following groups: 0 to 4, 5 to 9, 10 to 17, and 18 to 21 years of age; 2) presence of CHD, subclassified as: no CHD, biventricular repair, atrial baffle, Fontan palliation, pre-Fontan palliation, and unoperated; 3) arrhythmia mechanism; 4) ablation target anatomic location, subclassified according to our prior work;^19^ 5) history of prior ablation (referred to as “repeat ablations”); 6) ablation energy source: radiofrequency only, Cryo only, radiofrequency and Cryo; 7) presence of multiple ablation targets; 8) anesthesia strategy; 9) elective case status; and 10) presence of a second attending, as documented on the IMPACT form.

### Statistical analysis

Descriptive statistics were calculated using standard parametric and nonparametric statistics as indicated. Case characteristics were compared across center and operator volume strata. We first compared the distribution of unadjusted outcomes across center and operator volume strata using the chi-square test or analysis of variance. Adjusted analyses were then made using multivariable logistic regression adjusting for the covariates. Models were created for the primary outcome of acute ablation success at the primary target. With the potentially complicated relationship between center and operator volume, we elected to create 2 separate models were for center and operator volume. As a sensitivity analysis, we also modeled acute ablation success at all targets. Restricted cubic splines were also used to evaluate for a nonlinear association between case volume and acute ablation success.

To evaluate whether the association between case volume and acute ablation success differed based on the intrinsic complexity of different ablation substrates, we performed subanalyses in the following groups: 1) concealed accessory pathway, first-time ablation, no CHD; 2) manifest accessory pathway/WPW, first-time ablation, no CHD; 3) atrioventricular nodal reentrant tachycardia, first-time ablation, no CHD; 4) atrial tachycardia, first-time ablation, no CHD; 5) premature ventricular contraction (PVC)/VT, first-time ablation, no CHD; 6) repeat ablation for supraventricular tachycardia/WPW; 7) ablation in CHD. Multivariable models were calculated for each of these subgroups.

Based on an unexpected observation regarding the effect of the presence of a second attending on acute ablation success, we performed additional analyses. Centers were classified according to typical staffing of cases. Based on visual inspection of the data, we defined 53 centers as “single-operator centers,” with a second operator present for < 30% of cases; we defined 21 centers as “two-operator centers,” with an additional operator present in more than 30% of cases (Supplemental Figure 4). Univariable analyses were performed as described previously. All analysis was performed with SAS (9.4).

## Results

### Analysis of center volume

Of the 18,666 cases included in the center volume analysis, 12.4% (2,312) were performed at low volume centers, 40.5% (7,563) at medium, 34.4% (6,419) at high, and 12.7% (2,372) at very-high volume centers (Table 1). Very high centers performed more ablations in patients at both ends of the age spectrum (age 0-4 years and age 18-21 years). Repeat ablations were most common at very-high centers. Very-high centers also had the highest frequency of patients with CHD.

**Table 1:** Center Volume: Demographics and Case Characteristics

	Total (N = 18,666)	Low (n = 2,312, 12.4%)	Medium (n = 7,563, 40.5%)	High (n = 6,419, 34.4%)	Very High (n = 2,372, 12.7%)	*P* Value
Demographics						
Age (y)						
0 to 4	503 (2.7%)	42 (1.8%)	167 (2.2%)	203 (1.0%)	91 (3.8%)	<0.01
5 to 9	2,676 (14.3%)	351 (15.2%)	1,095 (14.5%)	900 (14.0%)	330 (13.9%)	
10 to 17	12,769 (68.4%)	1,587 (68.6%)	5,263 (69.6%)	4,394 (68.5%)	1,525 (64.3%)	
18 to 21	2,716 (14.6%)	332 (14.4%)	1,036 (13.7%)	922 (14.4%)	426 (18.0%)	
Weight (kg)	58.2 ± 24.6	58.4 ± 24.3	58.8 ± 25.0	57.9 ± 24.4	57.1 ± 23.4	0.02
Male	9,732 (52.1%)	1,200 (52.0%)	3,978 (52.6%)	3,301 (51.4%)	1,253 (52.8%)	0.48
Case characteristics						
Primary arrhythmia mechanism						
AP-concealed	3,902 (21%)	507 (22%)	1,670 (22%)	1,265 (20%)	460 (20%)	0.01
WPW	6,564 (36%)	826 (36%)	2,594 (34%)	2,265 (35%)	878 (37%)	0.36
AVNRT	5,334 (29%)	640 (28%)	2,217 (29%)	1871 (29%)	606 (26%)	0.01
Atrial tachycardia	1833 (10%)	209 (9%)	668 (9%)	653 (10%)	303 (13%)	<0.01
Ventricular tachycardia	825 (4%)	88 (4%)	343 (5%)	298 (5%)	96 (4%)	0.47
Atriofascicular pathway∗	129 (1%)	19 (1%)	38 (1%)	45 (1%)	27 (1%)	0.03
Cryo used	5,523 (30%)	579 (26%)	2,167 (29%)	2,101 (33%)	676 (29%)	<0.01
CHD	1,771 (9%)	154 (7%)	655 (8%)	632 (9%)	330 (14%)	<0.01
Repeat ablation	2,327 (12%)	265 (11%)	974 (13%)	739 (11%)	349 (15%)	<0.01

AP = accessory pathway; AVNRT = atrioventricular nodal reentrant tachycardia; CHD = congenital heart disease; WPW = Wolff-Parksinson-White.

∗ Atriofascicular pathways (unidirectional antegrade decremental pathways) were excluded from the analysis given rarity of substrate.

In univariable analysis of ablation outcomes, there was no statistically significant difference for ablation success at the primary target when compared across the 4 volume categories (Table 2). The highest rates of acute success at secondary and tertiary targets were among high centers. The median procedure times decreased with increasing volume, with shortest case times in very-high centers. Major adverse events were rare and not associated with volume. Rates of any adverse event were lowest (3%) in high volume centers.

**Table 2:** Ablation Outcomes

	Total (N = 18,666)	Low (n = 2,317)	Medium (n = 7,563)	High (n = 6,419)	Very High (n = 2,372)	*P* Value
Acute ablation success by target						
Primary	17,204 (92%)	2,109 (91%)	6,972 (91%)	5,940 (92%)	2,183 (92%)	0.24
Secondary	1,502 (85%)	161 (81%)	669 (84%)	481 (89%)	191 (82%)	0.01
Tertiary	149 (83%)	19 (70%)	65 (83%)	52 (89%)	13 (76%)	0.15
All targets	17,603 (92%)	2,098 (90%)	6,908 (91%)	5,901 (92%)	2,156 (91%)	0.22
Procedure time (hours)	2.8 (2.1-3.9)	3.1 (2.2-4.3)	2.84 (2.1-3.9)	2.7 (1.9-3.8)	2.5 (2.0-3.4)	<0.01
Adverse events						
Major	34 (0.1%)	3 (0.1%)	16 (0.2%)	13 (0.2%)	2 (0.1%)	0.56
Any	753 (4%)	125 (5%)	340 (4%)	180 (3%)	108 (5%)	<0.01

**Figure 3:**
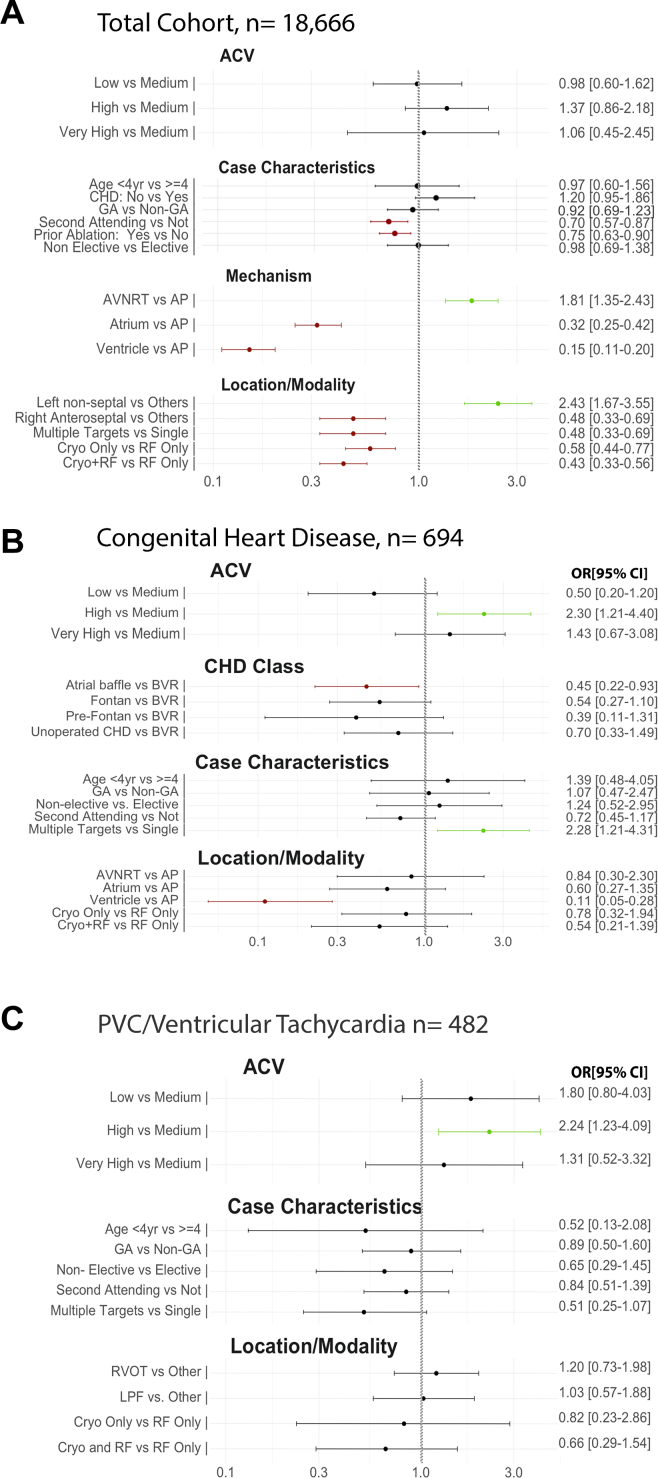
**Multivariable Models of Acute Success at Primary Target** Multivariable modeling of correlates of acute success including center ACV. (A) Includes all available cases, assessing predictive factors for acute success at the primary target. (B) Includes all cases of patient with congenital heart disease and (C) includes patients with ventricular substrates. AP = accessory pathway; AVNRT = atrioventricular nodal reentrant tachycardia; BVR = biventricular repair; CHD = congenital heart disease; Cryo = cryotherapy; GA = general anesthesia; LPF = left posterior fascicle; PVC = premature ventricular contraction; RF = radiofrequency; RVOT = right ventricular outflow tract.

In multivariable analysis, center volume was not significantly associated with acute ablation success at the primary target (Figure 3A). Subanalyses demonstrated increased odds of success among high volume centers in patients with CHD (OR: 2.30 [1.21-4.40]; *P* < 0.01), and VT or PVCs (OR: 2.2 [1.2-4.1]; *P* < 0.01) (Figures 3B and 3C). Among patients with CHD, multiple targets were interestingly associated with increased odds of acute success (OR: 2.3 [1.2-4.3]). Significant correlates of acute success are summarized in Table 3. Subanalyses among more common substrates including accessory pathways, atrioventricular nodal reentrant tachycardia, and atrial tachycardias, as well as analyses including an outcome of success at all targets, failed to show an association of center volume with success (Supplemental Tables 2 and 3).

**Table 3:** Significant Correlates of Acute Success (N = 18,666)

	Correlates (Reference)	OR (95% CI)
Case characteristics	Second attending present (vs not)	0.76 (0.57-0.87)
	Repeat ablation (vs no prior ablation)	0.75 (0.63-0.90)
Mechanism	AVNRT (vs AP)	1.81 (1.35-2.43)
	Atrial (vs AP)	0.32 (0.25-0.42)
	Ventricular (vs AP)	0.15 (0.11-0.20)
Location/modality	Left nonseptal (vs others)	2.43 (1.67-3.55)
	Right anteroseptal (vs others)	0.48 (0.33-0.69)
	Multiple targets (vs single)	0.48 (0.33-0.69)
	Cryo only (vs RF only)	0.58 (0.44-0.77)
	Cryo + RF (vs RF only)	0.43 (0.33-0.56)
Congenital heart disease subgroup (n = 694)		
ACV	High (vs medium)	2.30 (1.21-4.40)
CHD class	Atrial baffle (vs BVR)	0.45 (0.22-0.93)
Case characteristics	Multiple targets (vs single)	2.28 (1.21-4.31)
Mechanism	Ventricular (vs AP)	0.11 (0.05-0.28)
PVC/ventricular tachycardia subgroup (n = 482)		
ACV	High (vs medium)	2.24 (1.23-4.09)

ACV = annualized case volume; BVR = biventricular repair; PVC = premature ventricular contraction; RF = radiofrequency; other abbreviations as in Table 1.

A second attending participated in 5,671 (30.5%) of cases included in the center analysis, of which 1,411 (25.0%) were performed at “single-operator centers.” When compared to cases with 2 attendings at “two-operator centers,” double staffed cases in “single-operator centers” were significantly more likely to include patients with CHD (26.0% vs 8.9%; *P* < 0.001), repeat ablations (42.0% vs 27.5%; *P* < 0.001), age <4 years (4.5% vs 3.2%; *P* < 0.001) or age 18 to 21 years (28.3% vs 12.7%, *P* < 0.001), and had lower rates of success (83.6% vs 91.1%; *P* < 0.001) and higher rates of adverse events (8.7% vs 3.0%; *P* < 0.001).

Cubic spline analysis of annualized case volume and probability of acute success at the primary target (Supplemental Figure 3) suggested a trend toward a nonlinear association but with overlapping CIs, indicating a small effect size that is unlikely to be clinically meaningful.

### Analysis of operator volume

Of the 18,144 cases included in the operator analysis, 1,316 (7.2%) were performed by low volume operators, 9,411 (51.2%) by medium, 4,608 (25.4%) by high, and 2,809 (15.5%) by very-high volume operators (Table 4). Notable differences in patient characteristics by operator volume included a higher percentage of age 18 to 21 and CHD in very-high operators.

**Table 4:** Operator Volume: Demographics and Case Characteristics

Ablation Counts	Total (N = 18,144)	Low (n = 1,316, 7%)	Medium (n = 9,411, 51%)	High (n = 4,608, 25%)	Very High (n = 2,809, 15%)	*P* Value
Demographics						
Age (y)						
0-4	406 (2%)	16 (1%)	178 (2%)	121.3 (3%)	79 (3%)	<0.01
5-9	2,131 (12%)	134 (10%)	1,173 (12%)	553 (12%)	330 (10%)	
10 to 17	13,570 (75%)	998 (76%)	7,120 (76%)	3,460 (75%)	1,992 (71%)	
18-21	2037 (11%)	168 (13%)	940 (10%)	474 (10%)	455 (16%)	
Weight (kg)	58.1 ± 24.4	59.4 ± 24.4	57.6 ± 24.0	57.8 ± 24.5	59.6 ± 25.6	<0.01
Male	9,438 (52%)	703 (53%)	4,847 (52%)	2,453 (53%)	1,435 (51%)	0.13
Case characteristics						
Primary arrhythmia mechanism						
AP-concealed	3,818 (21%)	310 (24%)	1,972 (21%)	1,041 (23%)	495 (18%)	0.04
WPW	6,559 (36%)	476 (36%)	3,554 (38%)	1,582 (34%)	947 (34%)	0.19
AVNRT	5,221 (30%)	380 (30%)	2,690 (29%)	1,324 (29%)	88 (28%)	<0.01
Atrial tachycardia	1,688 (9%)	110 (8%)	774 (8%)	420 (9%)	384 (14%)	<0.01
Ventricular tachycardia	781 (4%)	35 (3%)	374 (4%)	233 (5%)	139 (5%)	0.07
Atriofascicular pathway^a^	129 (1%)	16 (1%)	71 (1%)	25 (1%)	17 (1%)	0.71
Cryo used	5,367 (30%)	357 (28%)	2,604 (28%)	1,296 (28%)	1,110 (40%)	<0.01
CHD	1,526 (8%)	10 (8%)	694 (7%)	374 (8%)	358 (15%)	<0.01
Repeat ablation	2,232 (12%)	137 (10%)	1,057 (11%)	620 (10%)	418 (15%)	<0.01

Abbreviations as in Table 1.

a Atriofascicular pathways (unidirectional antegrade decremental pathways) were excluded from the analysis given rarity of substrate.

In univariable analysis of ablation outcomes, there were significant differences for ablation success at the primary target, with the highest rate of acute success among very-high volume operators (Table 5). There was a trend toward higher rates of success at all targets in very-high operators, but this did not reach statistical significance. The median procedure times were lowest among high volume operators. Major adverse events were rare and not associated with volume. Rates of any adverse event rate were lowest among high and medium volume operators (3%) (Table 5).

**Table 5:** Operator Volume: Ablation Outcomes

	Total (N = 18,144)	Low (n = 1,316, 7%)	Medium (n = 9,411, 51%)	High (n = 4,608, 25%)	Very High (n = 2,809, 16%)	*P* Value
Acute ablation success by target						
Primary	16,755 (92%)	1,216 (92%)	8,656 (92%)	4,248 (92%)	2,635 (94%)	0.015
Secondary	1,452 (85%)	83 (81%)	762 (85%)	323 (85%)	284 (89%)	0.19
Tertiary	142 (83%)	10 (77%)	75 (78%)	31 (91%)	26 (90%)	0.22
All targets	16,616 (91%)	1,209 (92%)	8,580 (91%)	4,222 (92%)	2,605 (93%)	0.069
Procedure time (hours)	2.8 (2.0-3.8)	3.2 (2.3-4.3)	3.0 (2.2-4.0)	2.4 (1.8, 3.3)	2.6 (2.0-3.6)	<0.01
Adverse events						
Major	34 (0.1%)	3 (0.1%)	16 (0.2%)	13 (0.2%)	2 (0.1%)	0.56
Any	716 (4%)	67 (5%)	318 (3%)	148 (3%)	183 (7%)	<0.01

**Figure 4:**
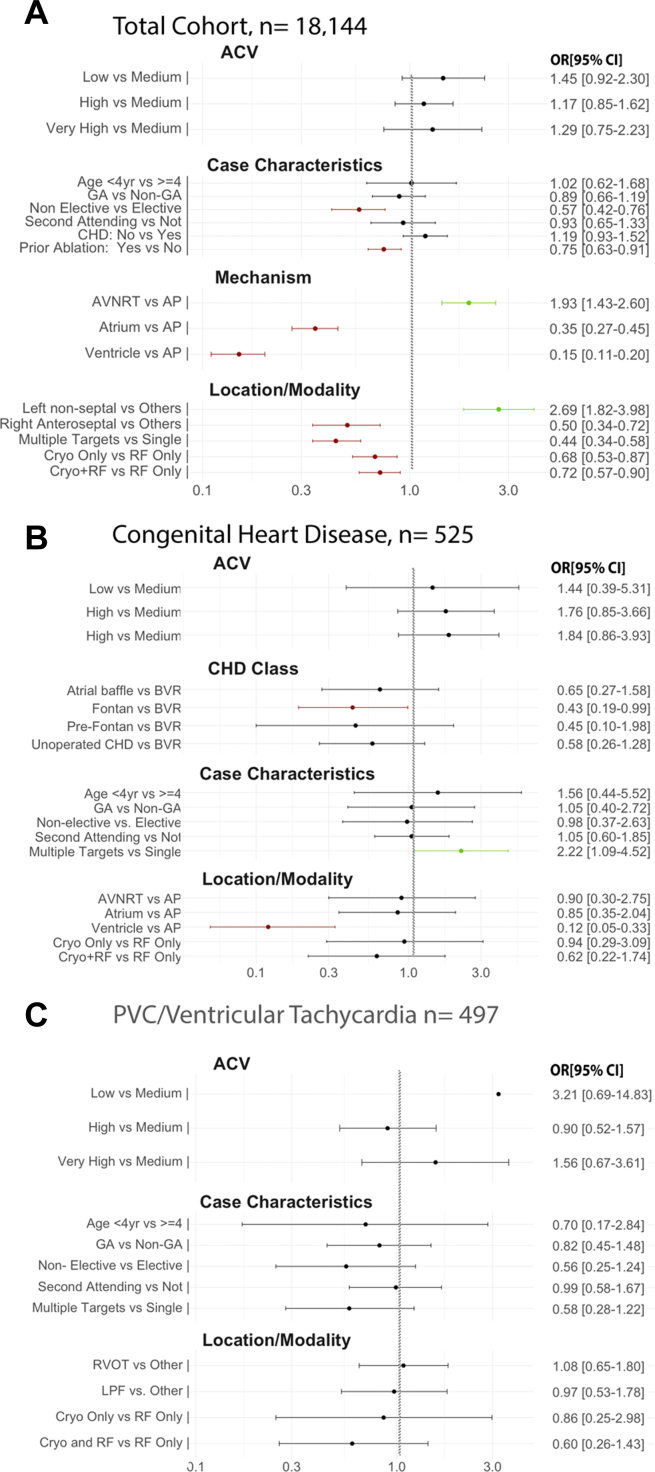
**Multivariable Models of Acute Success at Primary Target** Multivariable modeling of correlates of acute success including operator ACV. (A) Includes all available cases, assessing predictive factors for acute success at the primary target. (B) Includes all cases of patient with congenital heart disease and (C) Includes patients with ventricular substrates. Abbreviations as in Figure 3.

**Figure 5:**
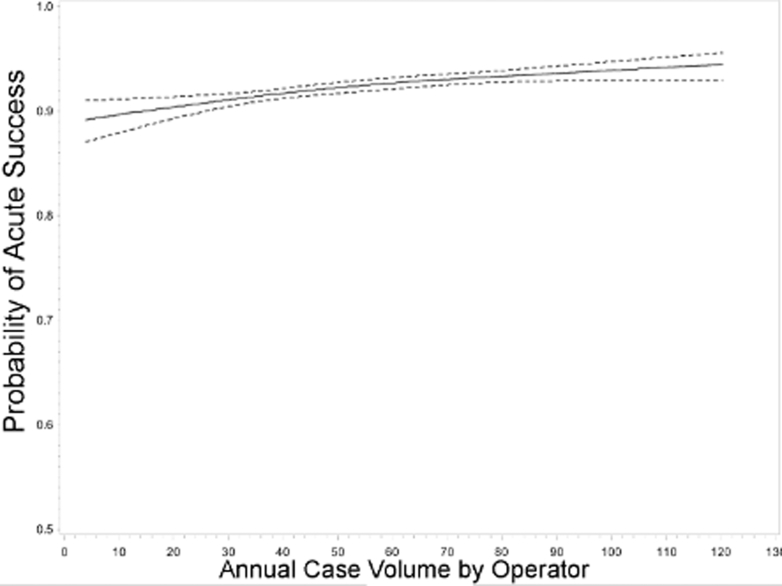
**Probability of Acute Success vs Annual Case Volume by Operator** Cubic spline analysis of the probability of success related to the annual case volume by operator. The dotted lines represent the 95% CI of the estimate.

In multivariable analysis, operator volume was not significantly associated with acute ablation success at the primary target (Figure 4A). Subanalyses failed to show any association of operator volume with ablation success, including among CHD and PVC/VT patients (Figures 4B and 4C). Significant correlates of acute success are summarized in Table 6. Cubic spline analysis demonstrates a progressive, albeit modest increase in probability of acute success with increasing operator volume (Figure 5).

**Table 6:** Significant Correlates of Acute Success: Operator (N = 18,144)

	Correlate (Reference)	OR (95% CI)
Case characteristics	Second attending present (vs not)	0.75 (0.63-0.91)
	Nonelective (vs elective)	0.57 (0.42-0.76)
Mechanism	AVNRT (vs AP)	1.93 (1.43-2.60)
	Atrial (vs AP)	0.35 (0.27-0.45)
	Ventricular (vs AP)	0.15 (0.11-0.20)
Location/modality	Left nonseptal (vs others)	2.69 (1.82-3.98)
	Right anteroseptal (vs others)	0.50 (0.34-0.72)
	Multiple targets (vs single)	0.44 (0.34-0.58)
	Cryo only (vs RF only)	0.68 (0.53-0.87)
	Cryo + RF (vs RF only)	0.72 (0.57-0.90)
Congenital heart disease subgroup (n = 525)		
ACV	High (vs medium)	2.30 (1.21-4.40)
Case characteristics	Multiple targets (vs single)	2.22 (1.09-4.52)
CHD class	Fontan (vs BVR)	0.43 (0.19-0.99)
Mechanism	Ventricular (vs AP)	0.12 (0.05-0.33)

Abbreviations as in Tables 1 and 3.

## Discussion

In this large-scale multicenter retrospective cohort study utilizing IMPACT registry data from 18,000 ablations, we investigated the hypotheses that higher center and higher operator case volumes would be associated with improved outcomes in pediatric catheter ablation. Overall, we found that for the most common pediatric ablation substrates, success rates were high and adverse events rates were low, independent of center or operator volume. However, for less common and more complex substrates, such as ventricular arrhythmias and arrhythmias in CHD, center volume is a significant factor, with increased odds of acute ablation success at high volume centers.

The multivariable model found that high volume centers had an increased odds of acute ablation success for PVC/VT cases (OR: 2.2) and CHD cases (OR: 2.3); however, in the parallel analysis operator volume was not independently associated with success for these substrates. This dissociation suggests that center-level factors beyond individual operator experience may have additive effects in rare or complex arrhythmias. These factors may include the latest mapping and ablation technologies, nursing and allied staff with additional EP experience, collaboration and discussion with more operators before and following a case, preprocedural imaging workflows, and anesthesia strategies that promote arrhythmia inducibility. Together these findings suggest for a small subset of rare and/or complex arrhythmia substrates, there may be benefit to treatment at higher-volume centers with greater collective experience and infrastructure which confer benefit independent of individual operator volume.

Adverse events and mortality represent key metrics where volume-outcome relationships have been demonstrated in procedural literature. For example, in recent work from the National Cardiovascular Data Registry AFib Registry,^13^ which included an analysis of over 70,000 ablations with a median major adverse event rate of 1.0%, low center volume was associated with an increased risk of MAE. Among analyses of congenital cardiac catheterization from the IMPACT database,^14^^,^^16^ it was major adverse events and the more nuanced failure to rescue metric where differences were seen according to procedural volumes. Fortunately, adverse event rates in pediatric ablations are quite low, in contrast to these other examples. With only 34 (0.1%) major adverse events in the present analysis, we did not conduct a multivariable analysis to evaluate the effect of case volume on adverse event rates.

The next point to consider is whether a different outcome variable, with a higher variance, would be more significantly affected by case volume—namely, the outcome of long-term success (or freedom from recurrence). Unfortunately, due to the constraints of the data set which consists of self-reported data entered at the time of the procedure, we were unable to compare recurrence rates by case volume. There is precedent in the adult atrial fibrillation literature that operator volume affects freedom from recurrence.^21^, ^22^, ^23^ In the multicenter study by Sairaku et al. acute success of pulmonary vein isolation was achieved in 99.1% of patients; however, high operator volume was the only independent predictor of freedom from AF recurrence (HR: 1.73; 95% CI: 1.25-2.48; *P* = 0.002). Future work in pediatric ablation should focus on refining high-impact outcome measures, including long-term freedom from recurrence, which may be associated with case volume, and identifying characteristics of cases that may benefit from ablation at a higher-volume center.

The present data analysis confirms previously described factors associated with acute ablation success and failure. Both models found tachycardia mechanism and anatomic location to be significant correlates of acute success. Both models also demonstrated that presence of multiple targets (with the exception the CHD subanalysis) and repeat procedures are associated with increased odds of acute ablation failure. Consistency of these observations with prior reports lends validity to the present data.

One unexpected observation is the association of 2 operators with reduced odds of ablation success in the center volume analysis. This variable was not significant in the operator volume analysis. The relationship between operator and center volume is complicated, so there may be confounding factors that contribute to differences in these models. We did explore this observation and noted that the presence of a second attending is likely a marker of case difficulty in single operator centers. In other words, the second attending is “called in” due to an intrinsically challenging case. Our data demonstrate practice variation in the utilization of a second operator, with 28% of centers employing 2 attendings in more than 50% of cases. The impact of a standard two-operator model on outcomes, including success, safety, and efficiency, warrants further investigation, but this was beyond the scope of the current analysis.

Another significant finding is that case duration was significantly associated with both center and operator volume. high and very-high volume centers and operators had shorter case durations, compared to medium and low volume, albeit with a small magnitude of difference. This finding may reflect increasing efficiency with increasing volume.

Finally, the current report contributes to our understanding of the range of annual case volumes for operators and centers that contribute data to IMPACT. These numbers could potentially serve as benchmarks for quality improvement initiatives. For the most part, the distribution of case characteristics is similar amongst low, medium, high, and very-high volume centers, though the 3 very-high volume centers appear to be enriched for CHD and ACHD cases. Although not within the scope of this analysis, 1 question is whether there is a minimum number of cases for an operator or center to maintain proficiency.

### Study limitations

In addition to those already mentioned, we acknowledge the following limitations. Most pediatric catheter ablation procedures are elective, which introduces the possibility for selective referral, with individuals or centers performing ablations when conditions are favorable, either based on patient (eg, age, size, anatomy) or substrate characteristics. Furthermore, evaluating longitudinal trends in procedural volume was not feasible. The staggered enrollment of participating institutions into the registry results in a relatively short timeframe where all centers were concurrently submitting data, which would heavily confound any temporal volume analyses. Matching case-mix to operator or center could introduce confounding by indication. This is supported by the observed variability in case mix, with increasing proportions of complex substrates and CHD at higher volumes without a significant change in acute success rates. As a retrospective study of fixed sample size, type II error is possible, especially with relatively rare adverse events. Although there are well-described auditing procedures for the IMPACT registry, self-report may introduce variability in the reporting of equivocal adverse events and technical performance. More importantly, IMPACT does not contain data on late complications or recurrence. Although annual volume is the best available measure of procedural volume, it does not capture career-long experience, especially of individual operators, which could also introduce important confounding. In addition, all cases may not contribute to expertise in the same way, either based on procedural complexity or about treatment of specific lesions. Future studies evaluating the associations between either volume of specific strata or lesions with outcomes may be valuable. Unmeasured confounding (eg undefined heterogeneity of PVC/VT and CHD patients) is an inevitable issue.

## Conclusions

Although an association was not seen between center or operator annual case volume and the likelihood of technical success across the board, there appear to be potentially important associations between case volume and the outcomes of less common, more complex cases, such as PVC/VT and CHD ablations. These outcomes appeared driven by institutional rather than individual operator experience, suggesting the importance of programmatic infrastructure.

## Funding support and author disclosures

The specific analysis described in this paper was funded by the 10.13039/100005485American College of Cardiology and the NCDR. The proposed project and paper were reviewed by the IMPACT Research and Publications Committee. The funding agencies had no role in the drafting of the manuscript or influencing its content. The views expressed in this paper represent those of the author(s), and do not necessarily represent the official views of the NCDR or its associated professional societies identified at CVQuality.ACC.org/NCDR. The authors have reported that they have no relationships relevant to the contents of this paper to disclose.
