# Supplementary material for: Association of Case Volume With Ablation Outcomes in Children: Analysis of the NCDR IMPACT Registry
Source: JACC Adv. 2026 Jun 17;5(6):102846. doi: 10.1016/j.jacadv.2026.102846 (PMC13308252; doi:10.1016/j.jacadv.2026.102846)
Supplement: Supplemental_Material [file mmc1.pdf]

Supplementary

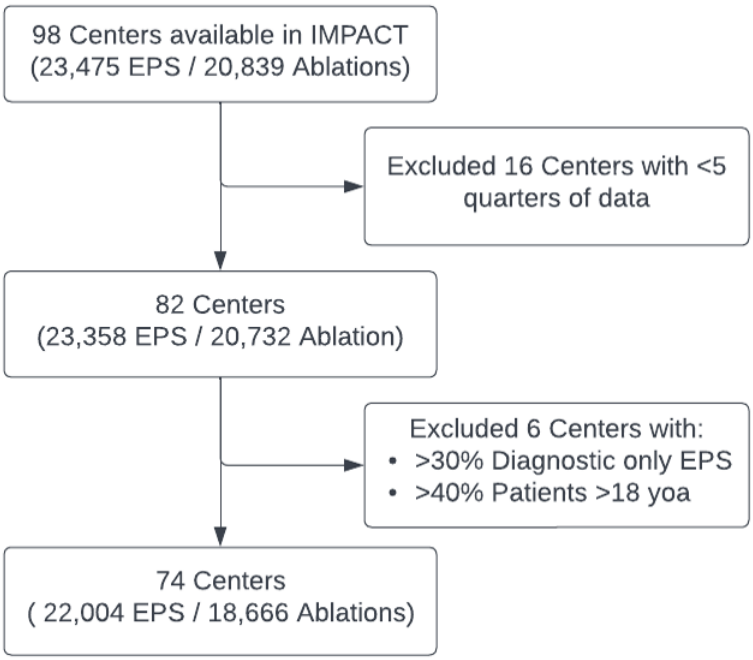

Supplementary Figure 1: Center Exclusion Diagram

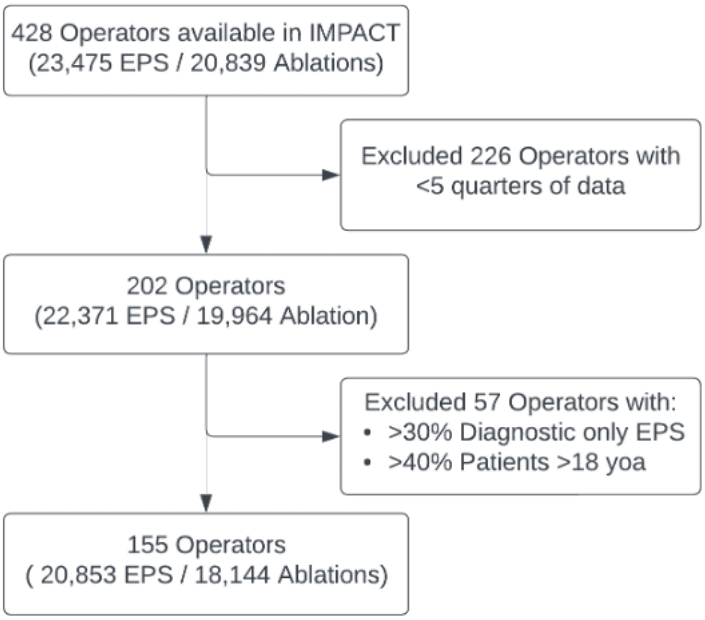

Supplementary Figure 2: Center Exclusion Diagram

Supplementary Table 1:

**Arrhythmia Substrate**	**Definition of Ablation Success**
Accessory pathway - Concealed	Elimination of retrograde AP conduction
Accessory pathway - Manifest (WPW) Bidirectional Antegrade only	Elimination of antegrade and retrograde conduction<br>Elimination of antegrade AP conduction
AVNRT AV node slow pathway modification	Elimination of slow pathway conduction<br>OR<br>Persistence of slow pathway conduction with single echos but no SVT<br>OR<br>Persistence of slow pathway conduction without echos
EAT	Substrate eliminated
PVC/VT	Elimination of spontaneous/inducible VT

Supplementary Figure 3: Cubic Spline analysis of probability of acute success correlated to annual center volume

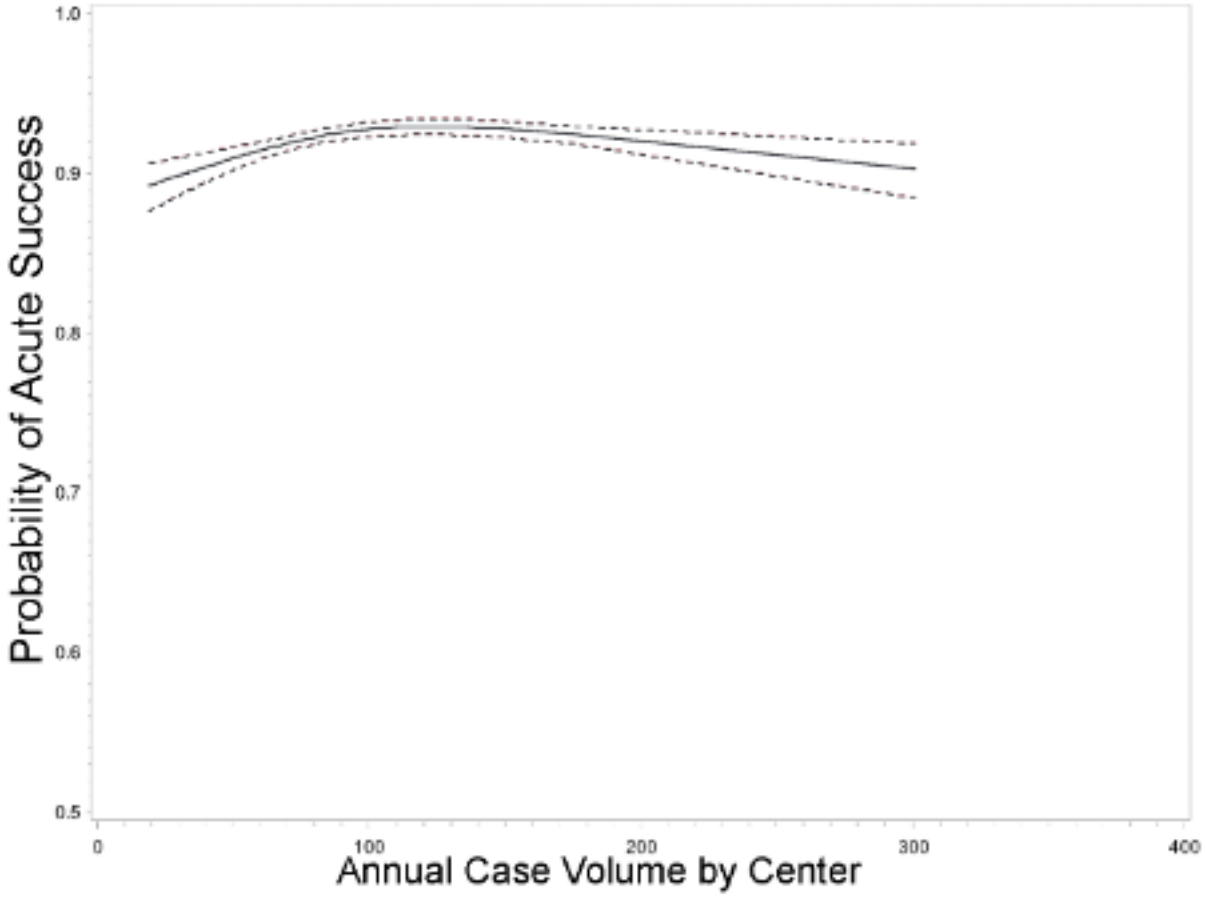

Supplementary Figure 4: Rates of Second Operator participation in analyzed cases by Center

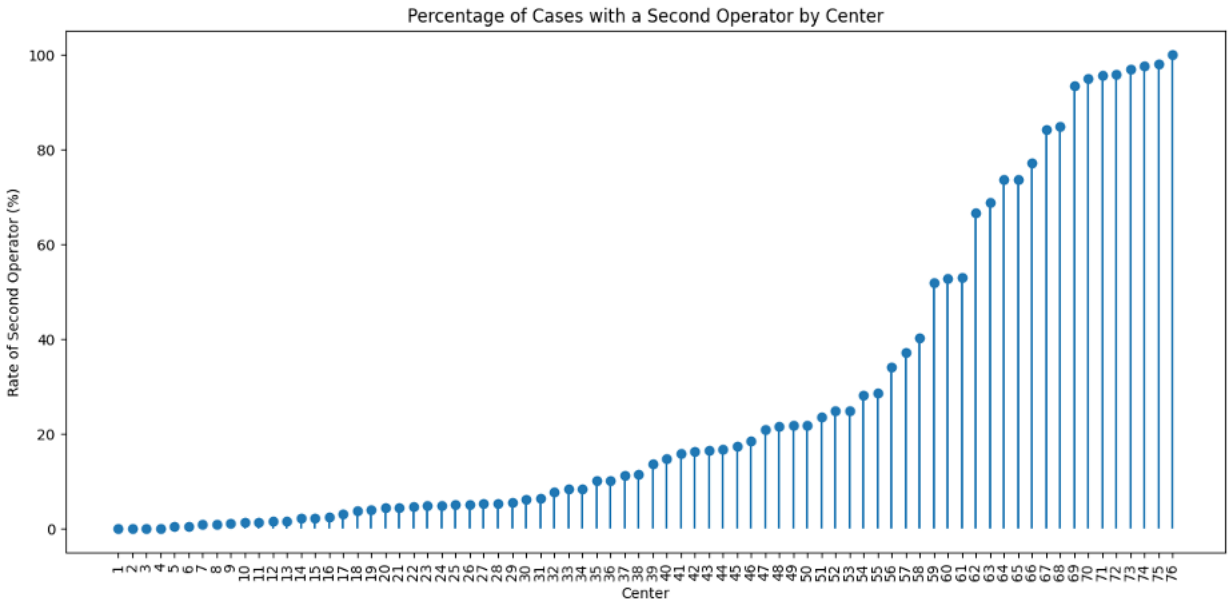

Supplementary Tables 2: Multivariable model of predictors of acute success including center volume:

a) Concealed Accessory pathway

Obs	Label	Odds Ratio for SUCCESS on Target1, 95% CI	Pvalue
**1**	Center Volume: Low vs Medium	0.83(0.41,1.69)	0.6095
**2**	Center Volume: High vs Medium	1.47(0.74,2.91)	0.2675
**3**	Center Volume: VeryHigh vs Medium	1.04(0.33,3.23)	0.9508
**4**	Age<4yr vs 4+	0.48(0.2,1.13)	0.0920
**5**	Location: Left non-septal vs Others	5.1(2.97,8.76)	<.0001
**6**	Location: Right Anteroseptal vs Others	0.23(0.13,0.41)	<.0001
**7**	GA vs Non-GA	1.26(0.65,2.46)	0.4958

Obs	Label	Odds Ratio for SUCCESS on Target1, 95% CI	Pvalue
**8**	Cryo Only vs RF Only	0.67(0.37,1.21)	0.1832
**9**	Cryo+RF vs RF Only	0.42(0.24,0.72)	0.0015
**10**	Non Elective vs Elective	1.4(0.31,6.25)	0.6589
**11**	Multiple Targets vs Single	0.32(0.2,0.52)	<.0001
**12**	SecondParticipating vs Not	0.68(0.42,1.11)	0.1234

b) WPW

Obs	Label	Odds Ratio for SUCCESS on Target1, 95% CI	Pvalue
**1**	Center Volume: Low vs Medium	0.66(0.39,1.13)	0.1273
**2**	Center Volume: High vs Medium	1.07(0.62,1.85)	0.8157
**3**	Center Volume: VeryHigh vs Medium	1.51(0.55,4.11)	0.4246
**4**	Age<4yr vs 4+	0.58(0.26,1.31)	0.1933
**5**	Location: Left non-septal vs Others	2(1.5,2.65)	<.0001
**6**	Location: Right Anteroseptal vs Others	0.39(0.27,0.56)	<.0001
**7**	GA vs Non-GA	1.12(0.76,1.63)	0.5755
**8**	Cryo Only vs RF Only	0.86(0.6,1.25)	0.4327
**9**	Cryo+RF vs RF Only	0.3(0.22,0.4)	<.0001
**10**	Non Elective vs Elective	1.05(0.49,2.25)	0.8994
**11**	Multiple Targets vs Single	0.67(0.48,0.92)	0.0144
**12**	SecondParticipating vs Not	0.69(0.51,0.91)	0.0099

c) AVNRT

Obs	Label	Odds Ratio for SUCCESS on Target1, 95% CI	Pvalue
1	Center Volume: Low vs Medium	0.67(0.31,1.43)	0.2991
2	Center Volume: High vs Medium	0.93(0.45,1.9)	0.8353
3	Center Volume: VeryHigh vs Medium	0.62(0.19,2.05)	0.4293
4	Age<4yr vs 4+	0.45(0.13,1.59)	0.2156
5	Location: RA Tri of Koch Posterio vs all-others	0.7(0.46,1.07)	0.0988
6	GA vs Non-GA	0.57(0.29,1.13)	0.1085
7	Cryo Only vs RF Only	0.47(0.28,0.78)	0.0034
8	Cryo+RF vs RF Only	0.52(0.27,0.97)	0.0414
9	Non Elective vs Elective	0.66(0.14,3.19)	0.6045
10	Multiple Targets vs Single	0.12(0.07,0.2)	<.0001
11	SecondParticipating vs Not	0.58(0.37,0.92)	0.0218

d) Repeat Ablations

Obs	Label	Odds Ratio for SUCCESS on Target1, 95% CI	Pvalue
1	Center Volume: Low vs Medium	0.8(0.36,1.75)	0.5701
2	Center Volume: High vs Medium	0.86(0.43,1.72)	0.6711
3	Center Volume: VeryHigh vs Medium	1.07(0.35,3.28)	0.9101
4	Location: LeftSide NonSeptal vs all-others	1.19(0.63,2.23)	0.5906
5	AVNRT vs AP (7 vs 1/2/3)	1.53(0.91,2.56)	0.1093
6	GA vs Non-GA	0.76(0.38,1.53)	0.4403
7	Cryo Only vs RF Only	0.52(0.3,0.89)	0.0169

Obs	Label	Odds Ratio for SUCCESS on Target1, 95% CI	Pvalue
**8**	Cryo+RF vs RF Only	0.39(0.24,0.66)	0.0004
**9**	Non Elective vs Elective	0.93(0.21,4.15)	0.9211
**10**	Multiple Targets vs Single	0.46(0.28,0.77)	0.0028
**11**	SecondParticipating vs Not	0.86(0.53,1.41)	0.5528

e) Atrial Tachycardia

Obs	Label	Odds Ratio for SUCCESS on Target1, 95% CI	Pvalue
**1**	Center Volume: Low vs Medium	0.64(0.33,1.25)	0.1905
**2**	Center Volume: High vs Medium	1.18(0.65,2.16)	0.5805
**3**	Center Volume: VeryHigh vs Medium	0.81(0.31,2.16)	0.6748
**4**	Age<4yr vs 4+	0.64(0.26,1.57)	0.3251
**5**	Target Right vs Left	0.73(0.47,1.13)	0.1532
**6**	GA vs Non-GA	1.29(0.79,2.11)	0.3044
**7**	Cryo Only vs RF Only	1.3(0.58,2.91)	0.5172
**8**	Cryo+RF vs RF Only	0.58(0.32,1.05)	0.0696
**9**	Non Elective vs Elective	0.89(0.49,1.61)	0.7045
**10**	Multiple Targets vs Single	0.73(0.4,1.34)	0.3126
**11**	SecondParticipating vs Not	0.63(0.39,1.01)	0.0562

Supplementary Tables 3: Multivariable model of predictors of acute success including operator volume:

a) Concealed Accessory Pathway

Obs	Label	Odds Ratio for SUCCESS on Target1, 95% CI	Pvalue
**1**	Operator Volume: Low vs Medium	0.84(0.41,1.73)	0.6298
**2**	Operator Volume: High vs Medium	1.28(0.72,2.27)	0.4069
**3**	Operator Volume: VeryHigh vs Medium	1.45(0.55,3.84)	0.4550
**4**	Age<4yr vs 4+	0.46(0.2,1.09)	0.0793
**5**	Location: Left non-septal vs Others	5.08(2.95,8.73)	<.0001
**6**	Location: Right Anteroseptal vs Others	0.24(0.13,0.43)	<.0001
**7**	GA vs Non-GA	1.28(0.65,2.52)	0.4753
**8**	Cryo Only vs RF Only	0.67(0.37,1.21)	0.1842
**9**	Cryo+RF vs RF Only	0.41(0.24,0.71)	0.0013
**10**	Non Elective vs Elective	1.34(0.3,5.98)	0.6996
**11**	Multiple Targets vs Single	0.32(0.19,0.52)	<.0001
**12**	SecondParticipating vs Not	0.76(0.46,1.23)	0.2639

b) WPW

Obs	Label	Odds Ratio for SUCCESS on Target1, 95% CI	Pvalue
**1**	Operator Volume: Low vs Medium	1.22(0.75,1.99)	0.4307
**2**	Operator Volume: High vs Medium	1.13(0.79,1.6)	0.5079
**3**	Operator Volume: VeryHigh vs Medium	1.69(0.85,3.39)	0.1359
**4**	Age<4yr vs 4+	0.58(0.26,1.31)	0.1927
**5**	Location: Left non-septal vs Others	1.97(1.48,2.62)	<.0001
**6**	Location: Right Anteroseptal vs Others	0.42(0.29,0.6)	<.0001
**7**	GA vs Non-GA	1.12(0.76,1.64)	0.5793
**8**	Cryo Only vs RF Only	0.83(0.57,1.2)	0.3154

Obs	Label	Odds Ratio for SUCCESS on Target1, 95% CI	Pvalue
**9**	Cryo+RF vs RF Only	0.29(0.21,0.39)	<.0001
**10**	Non Elective vs Elective	1.06(0.49,2.28)	0.8791
**11**	Multiple Targets vs Single	0.66(0.48,0.92)	0.0148
**12**	SecondParticipating vs Not	0.7(0.52,0.93)	0.0151

c) AVNRT

Obs	Label	Odds Ratio for SUCCESS on Target1, 95% CI	Pvalue
**1**	Operator Volume: Low vs Medium	0.86(0.41,1.79)	0.6865
**2**	Operator Volume: High vs Medium	1.19(0.67,2.13)	0.5548
**3**	Operator Volume: VeryHigh vs Medium	1.86(0.73,4.7)	0.1913
**4**	Age<4yr vs 4+	0.45(0.13,1.58)	0.2124
**5**	Location: RA Tri of Koch Posterio vs all-others	0.69(0.45,1.04)	0.0765
**6**	GA vs Non-GA	0.58(0.3,1.15)	0.1189
**7**	Cryo Only vs RF Only	0.45(0.27,0.75)	0.0021
**8**	Cryo+RF vs RF Only	0.5(0.27,0.95)	0.0331
**9**	Non Elective vs Elective	0.69(0.14,3.38)	0.6512
**10**	Multiple Targets vs Single	0.12(0.07,0.2)	<.0001
**11**	SecondParticipating vs Not	0.63(0.4,0.99)	0.0468

d) Repeat Ablations

Obs	Label	Odds Ratio for SUCCESS on Target1, 95% CI	Pvalue
**1**	Operator Volume: Low vs Medium	0.77(0.33,1.8)	0.5517

Obs	Label	Odds Ratio for SUCCESS on Target1, 95% CI	Pvalue
**2**	Operator Volume: High vs Medium	1.35(0.73,2.48)	0.3343
**3**	Operator Volume: VeryHigh vs Medium	1.47(0.62,3.48)	0.3763
**4**	Location: LeftSide NonSeptal vs all-others	1.2(0.64,2.25)	0.5725
**5**	AVNRT vs AP (7 vs 1/2/3)	1.53(0.91,2.57)	0.1052
**6**	GA vs Non-GA	0.79(0.39,1.58)	0.4995
**7**	Cryo Only vs RF Only	0.51(0.29,0.88)	0.0151
**8**	Cryo+RF vs RF Only	0.39(0.23,0.65)	0.0003
**9**	Non Elective vs Elective	0.95(0.21,4.27)	0.9509
**10**	Multiple Targets vs Single	0.47(0.29,0.79)	0.0039
**11**	SecondParticipating vs Not	0.89(0.55,1.44)	0.6281

e) Atrial Tachycardias

Obs	Label	Odds Ratio for SUCCESS on Target1, 95% CI	Pvalue
**1**	Operator Volume: Low vs Medium	0.85(0.39,1.86)	0.6858
**2**	Operator Volume: High vs Medium	1.04(0.59,1.84)	0.8832
**3**	Operator Volume: VeryHigh vs Medium	1.47(0.69,3.14)	0.3222
**4**	Age<4yr vs 4+	0.71(0.28,1.81)	0.4700
**5**	Target Right vs Left	0.71(0.46,1.1)	0.1300
**6**	GA vs Non-GA	1.36(0.83,2.23)	0.2230
**7**	Cryo Only vs RF Only	1.19(0.53,2.65)	0.6777
**8**	Cryo+RF vs RF Only	0.64(0.35,1.18)	0.1529

Obs	Label	Odds Ratio for SUCCESS on Target1, 95% CI	Pvalue
**9**	Non Elective vs Elective	0.86(0.46,1.59)	0.6299
**10**	Multiple Targets vs Single	0.68(0.37,1.27)	0.2241
**11**	SecondParticipating vs Not	0.73(0.45,1.19)	0.2082

Supplementary Table 3 – Details of Adverse Events by Center Volume

	Total	Low	Medium	High	Very High	
	n= 18,666	n = 2,317	n = 7,563	n = 6,419	n = 2,372	
any_adverse_event<br>1<br>0	 753 (4.02%)<br>17998 (95.98%)	 125 (5.39%)<br>2192 (94.61%)	 340 (4.45%)<br>7293 (95.55%)	 180 (2.80%)<br>6247 (97.20%)	 108 (4.55%)<br>2266 (95.45%)	< 0.001
major_adverse_event<br>1<br>0	 34 (0.18%)<br>18717 (99.82%)	 3 (0.13%)<br>2314 (99.87%)	 16 (0.21%)<br>7617 (99.79%)	 13 (0.20%)<br>6414 (99.80%)	 2 (0.08%)<br>2372 (99.92%)	0.557
Discharge Status<br>Alive<br>Deceased	 18744 (99.96%)<br>7 (0.04%)	 2317 (100.00%)<br>0 (0.00%)	 7630 (99.96%)<br>3 (0.04%)	 6424 (99.95%)<br>3 (0.05%)	 2373 (99.96%)<br>1 (0.04%)	0.791
Death in Lab<br>No<br>Missing	 7 (100.00%)<br>18744	 <br>2317	 3 (100.00%)<br>7630	 3 (100.00%)<br>6424	 1 (100.00%)<br>2373	
Cardiac Arrest<br>Yes<br>No<br>Missing	 20 (0.11%)<br>18693 (99.89%)<br>38	 1 (0.04%)<br>2309 (99.96%)<br>7	 10 (0.13%)<br>7604 (99.87%)<br>19	 8 (0.12%)<br>6410 (99.88%)<br>9	 1 (0.04%)<br>2370 (99.96%)<br>3	0.489
Arrhythmia<br>Yes<br>No<br>Missing	 331 (1.77%)<br>18383 (98.23%)<br>37	 82 (3.55%)<br>2228 (96.45%)<br>7	 117 (1.54%)<br>7497 (98.46%)<br>19	 90 (1.40%)<br>6328 (98.60%)<br>9	 42 (1.77%)<br>2330 (98.23%)<br>2	< 0.001
Arrhythmia Requiring Cardioversion<br>Yes<br>No	 117 (0.62%)<br>18634 (99.38%)	 21 (0.91%)<br>2296 (99.09%)	 46 (0.60%)<br>7587 (99.40%)	 37 (0.58%)<br>6390 (99.42%)	 13 (0.55%)<br>2361 (99.45%)	0.320
Arrhythmia Requiring Antiarrhythmic Medication<br>Yes<br>No	 91 (0.49%)<br>18660 (99.51%)	 18 (0.78%)<br>2299 (99.22%)	 35 (0.46%)<br>7598 (99.54%)	 24 (0.37%)<br>6403 (99.63%)	 14 (0.59%)<br>2360 (99.41%)	0.093
Arrhythmia Requiring Permanent Pacemaker<br>Yes<br>No	 7 (0.04%)<br>18744 (99.96%)	 0 (0.00%)<br>2317 (100.00%)	 2 (0.03%)<br>7631 (99.97%)	 2 (0.03%)<br>6425 (99.97%)	 3 (0.13%)<br>2371 (99.87%)	0.101
Arrhythmia Requiring Temporary Pacemaker<br>Yes<br>No	 9 (0.05%)<br>18742 (99.95%)	 2 (0.09%)<br>2315 (99.91%)	 1 (0.01%)<br>7632 (99.99%)	 3 (0.05%)<br>6424 (99.95%)	 3 (0.13%)<br>2371 (99.87%)	0.127
AV Block<br>Yes<br>No	 89 (0.47%)<br>18662 (99.53%)	 16 (0.69%)<br>2301 (99.31%)	 36 (0.47%)<br>7597 (99.53%)	 21 (0.33%)<br>6406 (99.67%)	 16 (0.67%)<br>2358 (99.33%)	0.064
New Heart Valve Regurgitation<br>No<br>Missing	 18713 (100.00%)<br>38	 2310 (100.00%)<br>7	 7614 (100.00%)<br>19	 6418 (100.00%)<br>9	 2371 (100.00%<br>)<br>3	

Tamponade<br>Yes<br>No<br>Missing	10 (0.05%)<br>18703 (99.95%)<br>38	3 (0.13%)<br>2307 (99.87%)<br>7	4 (0.05%)<br>7610 (99.95%)<br>19	3 (0.05%)<br>6415 (99.95%)<br>9	0 (0.00%)<br>2371 (100.00%<br>)<br>3	0.278
Air Embolus<br>Yes<br>No<br>Missing	2 (0.01%)<br>18711 (99.99%)<br>38	0 (0.00%)<br>2310 (100.00%)<br>7	1 (0.01%)<br>7613 (99.99%)<br>19	0 (0.00%)<br>6418 (100.00%)<br>9	1 (0.04%)<br>2370 (99.96%)<br>3	0.365
Embolic Stroke<br>Yes<br>No<br>Missing	1 (0.01%)<br>18712 (99.99%)<br>38	0 (0.00%)<br>2310 (100.00%)<br>7	0 (0.00%)<br>7614 (100.00%)<br>19	1 (0.02%)<br>6417 (99.98%)<br>9	0 (0.00%)<br>2371 (100.00%<br>)<br>3	0.590
Device Malposition or Thrombus<br>Yes<br>No<br>Missing	2 (0.01%)<br>18711 (99.99%)<br>38	0 (0.00%)<br>2310 (100.00%)<br>7	1 (0.01%)<br>7613 (99.99%)<br>19	1 (0.02%)<br>6417 (99.98%)<br>9	0 (0.00%)<br>2371 (100.00%<br>)<br>3	0.876
Device Embolization (Requiring device retrieval)<br>No<br>Missing	18713 (100.00%)<br>38	2310 (100.00%)<br>7	7614 (100.00%)<br>19	6418 (100.00%)<br>9	2371 (100.00%<br>)<br>3	
New Requirement for Dialysis<br>No<br>Missing	18713 (100.00%)<br>38	2310 (100.00%)<br>7	7614 (100.00%)<br>19	6418 (100.00%)<br>9	2371 (100.00%<br>)<br>3	
Coronary Artery Compression<br>Yes<br>No<br>Missing	3 (0.02%)<br>18710 (99.98%)<br>38	0 (0.00%)<br>2310 (100.00%)<br>7	1 (0.01%)<br>7613 (99.99%)<br>19	1 (0.02%)<br>6417 (99.98%)<br>9	1 (0.04%)<br>2370 (99.96%)<br>3	0.700
Erosion<br>Yes<br>No<br>Missing	1 (0.01%)<br>18712 (99.99%)<br>38	0 (0.00%)<br>2310 (100.00%)<br>7	0 (0.00%)<br>7614 (100.00%)<br>19	0 (0.00%)<br>6418 (100.00%)<br>9	1 (0.04%)<br>2370 (99.96%)<br>3	0.075
Esophageal Fistula<br>Yes<br>No<br>Missing	1 (0.01%)<br>18712 (99.99%)<br>38	0 (0.00%)<br>2310 (100.00%)<br>7	0 (0.00%)<br>7614 (100.00%)<br>19	0 (0.00%)<br>6418 (100.00%)<br>9	1 (0.04%)<br>2370 (99.96%)<br>3	0.075
Left Bundle Branch Block (LBBB)<br>Yes<br>No<br>Missing	6 (0.03%)<br>18707 (99.97%)<br>38	1 (0.04%)<br>2309 (99.96%)<br>7	5 (0.07%)<br>7609 (99.93%)<br>19	0 (0.00%)<br>6418 (100.00%)<br>9	0 (0.00%)<br>2371 (100.00%<br>)<br>3	0.133
Right Bundle Branch Block (RBBB)<br>Yes<br>No<br>Missing	47 (0.25%)<br>18666 (99.75%)<br>38	9 (0.39%)<br>2301 (99.61%)<br>7	25 (0.33%)<br>7589 (99.67%)<br>19	8 (0.12%)<br>6410 (99.88%)<br>9	5 (0.21%)<br>2366 (99.79%)<br>3	0.049
Airway Event Requiring Escalation of Care<br>Yes<br>No<br>Missing	7 (0.04%)<br>18706 (99.96%)<br>38	0 (0.00%)<br>2310 (100.00%)<br>7	4 (0.05%)<br>7610 (99.95%)<br>19	2 (0.03%)<br>6416 (99.97%)<br>9	1 (0.04%)<br>2370 (99.96%)<br>3	0.702
Event Requiring ECMO<br>Yes<br>No<br>Missing	3 (0.02%)<br>18710 (99.98%)<br>38	0 (0.00%)<br>2310 (100.00%)<br>7	1 (0.01%)<br>7613 (99.99%)<br>19	2 (0.03%)<br>6416 (99.97%)<br>9	0 (0.00%)<br>2371 (100.00%<br>)<br>3	0.635
Event Requiring LVAD<br>Yes<br>No<br>Missing	1 (0.01%)<br>18712 (99.99%)<br>38	0 (0.00%)<br>2310 (100.00%)<br>7	1 (0.01%)<br>7613 (99.99%)<br>19	0 (0.00%)<br>6418 (100.00%)<br>9	0 (0.00%)<br>2371 (100.00%<br>)<br>3	0.692
Bleeding Event<br>Yes<br>No<br>Missing	55 (0.29%)<br>18657 (99.71%)<br>39	9 (0.39%)<br>2300 (99.61%)<br>8	12 (0.16%)<br>7602 (99.84%)<br>19	20 (0.31%)<br>6398 (99.69%)<br>9	14 (0.59%)<br>2357 (99.41%)<br>3	0.005

RBC Transfusion<br>Yes<br>No<br>Missing	15 (0.08%)<br>18698 (99.92%)<br>38	2 (0.09%)<br>2308 (99.91%)<br>7	5 (0.07%)<br>7609 (99.93%)<br>19	6 (0.09%)<br>6412 (99.91%)<br>9	2 (0.08%)<br>2369 (99.92%)<br>3	0.948
Other Vascular Complications Requiring Treatment<br>Yes<br>No<br>Missing	11 (0.06%)<br>18697 (99.94%)<br>43	1 (0.04%)<br>2307 (99.96%)<br>9	6 (0.08%)<br>7606 (99.92%)<br>21	2 (0.03%)<br>6415 (99.97%)<br>10	2 (0.08%)<br>2369 (99.92%)<br>3	0.634
Other Events<br>Yes<br>No<br>Missing	113 (0.60%)<br>18591 (99.40%)<br>47	15 (0.65%)<br>2295 (99.35%)<br>7	53 (0.70%)<br>7556 (99.30%)<br>24	25 (0.39%)<br>6389 (99.61%)<br>13	20 (0.84%)<br>2351 (99.16%)<br>3	0.039
Peripheral Nerve Injury<br>Yes<br>No<br>Missing	5 (0.03%)<br>18707 (99.97%)<br>39	1 (0.04%)<br>2309 (99.96%)<br>7	1 (0.01%)<br>7612 (99.99%)<br>20	2 (0.03%)<br>6416 (99.97%)<br>9	1 (0.04%)<br>2370 (99.96%)<br>3	0.795
Phrenic Nerve Paralysis<br>No<br>Missing	18712 (100.00%)<br>39	2310 (100.00%)<br>7	7613 (100.00%)<br>20	6418 (100.00%)<br>9	2371 (100.00%<br>)<br>3	
Pneumothorax<br>Yes<br>No<br>Missing	2 (0.01%)<br>18710 (99.99%)<br>39	0 (0.00%)<br>2310 (100.00%)<br>7	1 (0.01%)<br>7612 (99.99%)<br>20	1 (0.02%)<br>6417 (99.98%)<br>9	0 (0.00%)<br>2371 (100.00%<br>)<br>3	0.876
Pulmonary Embolism<br>No<br>Missing	18712 (100.00%)<br>39	2310 (100.00%)<br>7	7613 (100.00%)<br>20	6418 (100.00%)<br>9	2371 (100.00%<br>)<br>3	
Pulmonary Vein Stenosis<br>Yes<br>No<br>Missing	1 (0.01%)<br>18643 (99.99%)<br>107	0 (0.00%)<br>2296 (100.00%)<br>21	1 (0.01%)<br>7563 (99.99%)<br>69	0 (0.00%)<br>6413 (100.00%)<br>14	0 (0.00%)<br>2371 (100.00%<br>)<br>3	0.690
Radiation Burn to Skin<br>Yes<br>No<br>Missing	1 (0.01%)<br>18711 (99.99%)<br>39	0 (0.00%)<br>2310 (100.00%)<br>7	0 (0.00%)<br>7613 (100.00%)<br>20	1 (0.02%)<br>6417 (99.98%)<br>9	0 (0.00%)<br>2371 (100.00%<br>)<br>3	0.590
Deep Vein Thrombosis<br>Yes<br>No<br>Missing	3 (0.02%)<br>18709 (99.98%)<br>39	2 (0.09%)<br>2308 (99.91%)<br>7	0 (0.00%)<br>7613 (100.00%)<br>20	1 (0.02%)<br>6417 (99.98%)<br>9	0 (0.00%)<br>2371 (100.00%<br>)<br>3	0.032
Conduit Tear<br>Yes<br>No<br>Missing	1 (0.01%)<br>18711 (99.99%)<br>39	0 (0.00%)<br>2310 (100.00%)<br>7	0 (0.00%)<br>7613 (100.00%)<br>20	0 (0.00%)<br>6418 (100.00%)<br>9	1 (0.04%)<br>2370 (99.96%)<br>3	0.075
Unplanned Cardiac Surgery<br>Yes<br>No<br>Missing	6 (0.03%)<br>18699 (99.97%)<br>46	0 (0.00%)<br>2307 (100.00%)<br>10	3 (0.04%)<br>7609 (99.96%)<br>21	3 (0.05%)<br>6412 (99.95%)<br>12	0 (0.00%)<br>2371 (100.00%<br>)<br>3	0.559
Unplanned Vascular Surgery<br>Yes<br>No<br>Missing	4 (0.02%)<br>18704 (99.98%)<br>43	0 (0.00%)<br>2310 (100.00%)<br>7	4 (0.05%)<br>7608 (99.95%)<br>21	0 (0.00%)<br>6415 (100.00%)<br>12	0 (0.00%)<br>2371 (100.00%<br>)<br>3	0.120
Unplanned Other Surgery<br>Yes<br>No<br>Missing	6 (0.03%)<br>18697 (99.97%)<br>48	0 (0.00%)<br>2308 (100.00%)<br>9	1 (0.01%)<br>7608 (99.99%)<br>24	4 (0.06%)<br>6411 (99.94%)<br>12	1 (0.04%)<br>2370 (99.96%)<br>3	0.320
Subsequent Cardiac Cath<br>Yes<br>No<br>Missing	11 (0.06%)<br>18694 (99.94%)<br>46	0 (0.00%)<br>2310 (100.00%)<br>7	3 (0.04%)<br>7606 (99.96%)<br>24	5 (0.08%)<br>6410 (99.92%)<br>12	3 (0.13%)<br>2368 (99.87%)<br>3	0.251

Cardiac Surgery during this admission						< 0.001
Yes	214 (1.15%)	8 (0.35%)	138 (1.82%)	36 (0.56%)	32 (1.35%)	
No	18469 (98.85%)	2300 (99.65%)	7444 (98.18%)	6383 (99.44%)	2342 (98.65%)	
Missing	68	9	51	8		
